# Observational study of predictors and outcomes of lung cancer in never-smokers in the UK (OLIVE): study protocol

**DOI:** 10.1136/bmjresp-2025-003966

**Published:** 2026-06-09

**Authors:** Sindhu Bhaarrati Naidu, Gabrielle Guvensen, Honghan Wu, Rumana Z Omar, Usha Menon, Sanjay Popat, David A Moore, Yiwen Soo, Amyn Bhamani, Ricky Thakrar, Tanya Ahmad, OLIVE PPI Group, Sam M Janes, Martin D Forster, Neal Navani

**Affiliations:** 1 UCL Respiratory, University College London Lungs for Living Research Centre, London, England, UK; 2 University College London, London, UK; 3 School of Health and Wellbeing, University of Glasgow, Glasgow, UK; 4 Institute of Health Informatics, University College London, London, UK; 5 Department of Statistical Science, UCL, London, UK; 6 MRC Clinical Trials Unit at UCL, Institute of Clinical Trials & Methodology, UCL, London, UK; 7 Lung Unit, The Royal Marsden Hospital, London, England, UK; 8 Division of Clinical Studies, The Institute of Cancer Research, London, England, UK; 9 CRUK Lung Cancer Centre of Excellence, UCL Cancer Institute, London, UK; 10 Department of Cellular Pathology, University College London Hospitals, London, England, UK; 11 Lungs for Living Research Centre, UCL Respiratory, University College London, London, UK; 12 Department of Medical Oncology, Cancer Division, University College London Hospitals, London, UK

**Keywords:** Clinical Epidemiology, Lung Cancer, Lung Cancer Chemotherapy, Non-Small Cell Lung Cancer

## Abstract

**Introduction:**

Lung cancer is commonly associated with smoking. However, if considered separately, lung cancer in never-smokers (LCINS) is the seventh most common cause of cancer-related death worldwide. Expanding the limited understanding of LCINS, especially in the UK, is crucial to improving diagnosis.

**Methods and analysis:**

We will establish a retrospective and prospective UK multicentre observational cohort comprising never-smoking (lifetime use of <100 tobacco cigarettes) adults with lung cancer using routinely available primary and secondary care electronic health records (EHRs). Demographic data including occupation and ethnicity, exposures and lung cancer outcomes will be extracted. Deep learning-based natural language processing will be used to analyse free-text data. Quantitative or qualitative data collection for information not available in EHR may be initiated in the future. Follow-up will be until death, withdrawal or end of study (5 years from study activation date). Primary outcomes will be sociodemographic characteristics, comorbidities, environmental exposures, pathways to presentation and symptoms. A feasibility pilot of 225 patients (25 participants per year both retrospectively and prospectively) will be undertaken at one hospital before extending the study to multiple sites. Patient representatives have been involved in adapting the research protocol including the recruitment process and patient-facing materials.

**Ethics and dissemination:**

National ethical approval has been obtained from Health Research Authority (HRA) (24/YH/0147). Access to patient records without explicit consent, including deceased patient records, is legally supported by the Confidentiality Advisory Group and HRA. The study is sponsored by University College London Hospital.

Data will be anonymised and analysed 2 years after the study start date and on study completion. Together with our patient representatives, we will disseminate data to the scientific and academic community and public through peer-reviewed publications, presentations at conferences, patient-facing charities and social media.

**WHAT IS ALREADY KNOWN ON THIS TOPIC**

**WHAT THIS STUDY ADDS**

- We will undertake a feasibility pilot in a single centre before expanding nationally to ensure representativeness of our cohort and provide sufficient power to answer research questions on characteristics and outcomes of participants with LCINS.

**HOW THIS STUDY MIGHT AFFECT RESEARCH, PRACTICE OR POLICY**

- Establishing a cohort of never-smokers with lung cancer creates opportunities for raising patient-centred research questions, testing early detection algorithms, and additional prospective data collection.
- Improving our understanding of risk of lung cancer in people who have never smoked, for example by understanding patient pathways and symptoms, may improve clinical practice and support guideline development.
- Increased awareness that lung cancer can occur in people who have never smoked will support earlier diagnosis.

## Introduction

 Lung cancer causes the most cancer deaths, both worldwide and in the UK. In 2022, 2 480 675 adults were diagnosed with and 1 817 469 people died from lung cancer worldwide; in the UK, 50 700 adults were diagnosed with and 35 394 people died from lung cancer.^1^ Only 48% of adults survive more than 1 year, which is likely related to late diagnosis: 45% of all patients are diagnosed with stage IV (the worst stage) at presentation and 32% diagnosed following an emergency presentation.^2^ Adults with lung cancer also have poorer quality of life and patient experience, with 33.7% having three or more primary care consultations before being referred, a higher proportion compared with most cancers.^3^

Although commonly associated with smoking, up to 25% of lung cancer worldwide occurs in ‘never-smokers’ or people who have smoked <100 tobacco cigarettes in their lifetime.^4^ If considered separately, lung cancer in never-smokers (LCINS) is the seventh most common cause of cancer death worldwide.^4^

Adults who have never smoked may have different risk factors, symptoms, genetic mutations and outcomes. Consequently, there is limited understanding of LCINS, especially in the UK, where it has been identified as a research priority.^5^ There is likely to be an association with sociodemographic characteristics including deprivation and female sex.^6^ As per the National Cancer Equality Initiative, understanding sociodemographic differences is important in addressing inequalities and improving outcomes.^7^ Individuals who have never smoked may also experience late diagnosis and stigmatisation; both healthcare professionals and health-seeking individuals may not consider the diagnosis of lung cancer due to perceived low risk.^8^ In addition, patient representatives have questioned if never-smokers present with the same symptoms as ever-smokers. There is also a paucity of data on outcomes in this cohort, with one UK-based study of 436 never-smokers only reporting on individuals presenting early enough to undergo surgical resection with curative intent.^9^

Reduction in lung cancer mortality and morbidity is therefore likely to be best achieved by facilitating early diagnosis.^10^ While lung cancer screening pilot programmes are now underway in the UK to achieve this goal, never-smokers are excluded from these programmes and improved outcomes associated with stage shift from screening will not be seen in never-smokers.^11^ More research is required to understand how to improve early diagnosis in this cohort. Improving understanding of LCINS is a necessary first step.

## Methods and analysis

OLIVE is an observational cohort study which will collect data retrospectively and prospectively on never-smoking participants with lung cancer predominantly using data routinely available from electronic health records (EHRs). It has been registered with clinicaltrials.gov (NCT06575439). The study will be initiated as a pilot in one hospital in North London (University College London Hospital (UCLH)), to understand the feasibility of data collection, including completeness of data items in EHR, and linkage. External funding will be sought to subsequently expand the study across multiple sites nationally to ensure representativeness of data. This study has been externally peer reviewed and adapted following feedback from an independent multidisciplinary committee including clinicians and patient representatives organised by the National Cancer Research Institute.

### Primary objectives

To establish a data lake of never-smokers with lung cancer to:

1. Describe characteristics of never-smokers with lung cancer including sociodemographic characteristics, comorbidities and environmental exposures.
2. Describe pathways to presentation and symptoms.

### Secondary objectives

1. Describe lung cancer outcomes such as stage at diagnosis, treatment and survival.
2. Quantitatively or qualitatively measure the association of modifiable and non-modifiable factors that may be associated with LCINS including environmental measures, blood tests and genomic data.

### Inclusion criteria

- Radiological or histological diagnosis of primary lung cancer.
- Smoked <100 tobacco cigarettes in their lifetime.
- Age over 18 years.

### Exclusion criteria

- Received a prescription for nicotine replacement therapy.
- For prospective recruitment, unable to consent at time of study entry.

### Recruitment of participants and consent

**Figure 1:**
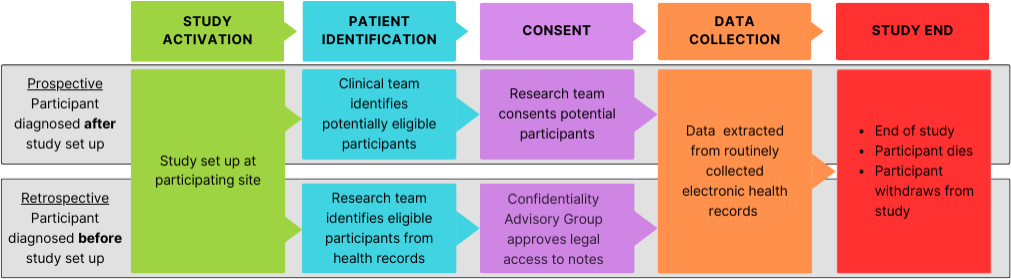
Participant recruitment process.

Participant recruitment is summarised in figure 1.

The study start/activation date is defined as the date the study goes live at UCLH.

Potential participants diagnosed after the study activation date at each site will be identified by a member of the clinical team during lung cancer multidisciplinary team meetings, clinics and via prescription lists for systemic therapy. Potential participants will be provided a patient information sheet (PIS) which will include contact details of a research team member. A research team member will contact them at least 24 hours later and provide an opportunity to ask any questions about the study. Participants may choose to speak either face-to-face or over the telephone. Non-English speakers will be spoken to with a translation service to ensure full understanding. If they consent, they will be asked to sign the consent form electronically or on paper. The consent process including PIS and consent form (online supplemental appendix 1) has been reviewed and modified by a team of patient representatives and the information provided was deemed to be ‘concise’, ‘extensive’ and ‘clear’.

National Lung Cancer Audit data suggest that 60% of patients diagnosed with lung cancer would have died at 1 year.^2^ Due to this high mortality rate, retrospective consent is unlikely to be feasible for all participants diagnosed before the study starts at each site. Participants diagnosed before the study activation date will therefore be identified from EHR and, unless part of National Data Opt Out, will be included in our research database. Two members of the research team will independently confirm never-smoking status by reviewing EHR and free-text clinical notes. This was discussed with patient representatives who felt this was acceptable and provided suggestions on how to increase awareness. A local opt-out option will be provided by displaying posters in clinical areas and by broadcasting on each site’s website, social media and patient portals. We have received legal approval from the confidentiality advisory group (CAG) and the Health Research Authority for this retrospective recruitment.

Participants will be followed up until death, withdrawal from the study or until the end of the study (5 years from study activation date at UCLH). Those who lose capacity will have ongoing EHR review but will not be invited for any extra data collection. This was discussed with patient representatives and thought to be ‘completely acceptable’ and is explained in our PIS.

### Data collection

Data will be collected on characteristics of never-smokers with lung cancer. Data to be collected were initially determined by a systematic review,^12^ with further variables included after feedback from patient representatives and experts in respiratory medicine and oncology. Data extraction will be trialled on 100 patients to review feasibility before finalising variables that will be collected.

Routinely collected data from primary and secondary care electronic health systems will include demographic data such as sex, occupation and ethnicity, comorbidities and cancer data and outcomes. Postcode at the time of data collection will be used to determine indices of deprivation and exposure to pollution. Investigations documented in EHR including blood tests, diagnostic imaging and cancer somatic data will be reviewed. Both primary and secondary care electronic notes will be reviewed to extract and analyse free-text data and data which are not otherwise coded such as symptoms leading to presentation. There will be opportunities for machine-learning based data analysis including natural language processing. In the future, further qualitative or quantitative data collection may be initiated for information not routinely available in EHR such as blood tests and pollution measurements.

### Data storage

Data will be stored in UCL Data Safe Haven, a secure solution for storing, handling and analysing identifiable data. This has been certified to ISO27001 information security standard and conforms to NHS Digital’s Information Governance Toolkit. All data will be stored, processed and managed within the security of this system. Data is protected in a ‘walled garden’ and users are prevented from accessing external network resources such as email. Data will be stored for 5 years after the end of the study.

### Sample size

It is estimated that our local hospital sees 400 patients with lung cancer each year. Between 10% and 20% (40–80) of these patients will be never-smokers. We believe it is feasible to recruit 25 participants per year both retrospectively (from 1 January 2019 to 2024 inclusive) and prospectively (from study start date in 2024 to 2029 inclusive), with an overall recruitment target of 225 participants. This will allow us to review feasibility of data collection and planned statistical analysis. We will then seek funding to expand the study across multiple sites to ensure sufficient power for data analysis. We will aim to choose appropriate sites across the UK to ensure the representativeness of the cohort.

### Data analysis

Interim analysis will be performed on the pilot cohort. Primary and secondary outcomes will be described using descriptive statistics including percentages for categorical variables and mean and SD or median and IQR for continuous variables. Groups will be compared such as using ANOVA for parametric, Mann-Whitney U for non-parametric and χ^2^ for categorical tests. All tests of significance will be two-tailed and a p value ≤0.05 will be considered statistically significant.

We will use machine learning algorithms such as deep-learning-based natural language processing (NLPs) to analyse free-text data to identify variables associated with LCINS including genomic data. UCLH has deployed information retrieval platforms with NLP capabilities such as CogStack^13^ and MedCAT.^14^ These models allow further adaptations for new datasets and tasks using annotated data. Data will be validated by manual annotation of at least 10% of participants.

Data may be analysed with Cox proportional hazards to determine the association of variables identified (such as exposures and presenting symptoms) with one-year and five-year survival as an outcome. We assume at least 100 outcome events are required to perform survival analysis involving 10 covariates. Given the high mortality associated with lung cancer, it is possible that this threshold will be met even within the pilot cohort. We will also review the possibility of extending follow-up of study participants if required.

Once variables to be collected have been finalised, we will review missingness of data including likely missingness mechanisms. If survival analysis is performed, where appropriate, multiple imputation based on chained equations will be used to impute missing values for predictor variables with more than 10% missingness. Sensitivity analyses including limiting analysis to complete cases only will be performed to assess the robustness of the results.

### Patient and public involvement

A clinical team caring for never-smokers with lung cancer identified suitable individuals to discuss this research. Four individuals agreed to take part in an online patient engagement meeting. They range in age from mid-30s to late-60s and represent different ethnic and socioeconomic groups. They have been diagnosed with lung cancer for different lengths of time and have different stages of disease. They were renumerated for their time and participation.

The research protocol was adapted following this discussion, including:

- Research design including which factors should be collected from EHR.
- The recruitment process including how to increase awareness of the opt-out process.
- The consent process including the PIS and consent form; for example, the term ‘never-smokers’ was defined in all patient-facing information.

We also discussed ethical considerations including their views on reviewing confidential data without explicit consent, and risks and benefits of the study. All representatives agreed there were no concerns and our research protocol is completely acceptable.

Our patient representatives will continue to engage with this research and ensure that the process remains acceptable to patients. They will also be involved in the analysis and dissemination including interpreting results and identifying research themes, reviewing plain English summaries and advising on the impact of results on the public. As discussed with our patient representatives, ongoing involvement will be through at least two 30-minute one-to-one meetings each year with each patient via Teams, Zoom or phone and minimum twice-yearly emails. Patient representatives have also agreed to additional group meetings for more collaborative discussions.

### Ethics and dissemination

The study is compliant with the requirements of General Data Protection Regulation (GDPR) (2016/679) and the UK Data Protection Act (2018). All investigators and study site staff will comply with the requirements of the GDPR (2016/679) with regards to the collection, storage, processing and disclosure of personal information and will uphold the Act’s core principles. Samples will be processed, stored and disposed in accordance with all applicable legal and regulatory requirements, including the Human Tissue Act 2004 and any amendments thereafter, and the applicable HTA Codes of Practice Departmental SOPs will be followed to facilitate regulatory compliance.

Ethical approval has been obtained from the Health Research Authority (HRA) (24/YH/0147). Access to patient records without explicit consent, including deceased patient records, is legally supported by the CAG (24CAG0077) and HRA. Any amendments to the protocol will be submitted for further approval. An annual review will be submitted to the CAG. The study is sponsored by UCLH.

As this is predominantly an observational study using data that are routinely collected within EHR, there will be no changes to usual clinical care and serious adverse events are not expected. All patient representatives thought the risk of being involved in the study was minimal.

Confidentiality and GDPR principles will be strictly adhered to. Risks to confidentiality will be minimised by requesting a minimally required dataset and processing to remove personal IDs where possible. All researchers have a duty of confidentiality and appropriate training.

Data will be anonymised and analysed two years after the study start date and on study completion. Only aggregated, anonymised data will be published. Data will be disseminated to the scientific and academic community through presentations at national and international conferences and publications. Together with our patient representatives, we will share our results with the public and patients through patient-facing charities, through social media and on hospital websites. Access to study data by external investigators can be requested by contacting the UCLH Joint Research Office and study Principal Investigator.

## Supplementary material

10.1136/bmjresp-2025-003966 online supplemental file 1

## Data Availability

Data are available upon reasonable request.

## References

[1] World Health Organization International agency for research on cancer. cancer today: data visualization tools for exploring the global cancer burden in 2020. http://gco.iarc.fr/today/home.

[2] (2024). National lung cancer audit state of the nation.

[3] Lyratzopoulos G, Neal RD, Barbiere JM (2012). Variation in number of general practitioner consultations before hospital referral for cancer: findings from the 2010 National Cancer Patient Experience Survey in England. Lancet Oncol.

[4] Sun S, Schiller JH, Gazdar AF (2007). Lung cancer in never smokers--a different disease. Nat Rev Cancer.

[5] Khan S, Hatton N, Tough D (2023). Lung cancer in never smokers (LCINS): development of a UK national research strategy. *BJC Rep*.

[6] Rait G, Horsfall L (2020). Twenty-year sociodemographic trends in lung cancer in non-smokers: A UK-based cohort study of 3.7 million people. Cancer Epidemiol.

[7] National Cancer Equality Initiative (2010). Reducing cancer inequality: evidence, progress and making it happen.

[8] Scott N, Crane M, Lafontaine M (2015). Stigma as a barrier to diagnosis of lung cancer: patient and general practitioner perspectives. Prim Health Care Res Dev.

[9] Cufari ME, Proli C, De Sousa P (2017). Increasing frequency of non-smoking lung cancer: Presentation of patients with early disease to a tertiary institution in the UK. Eur J Cancer.

[10] Barta JA, Powell CA, Wisnivesky JP (2019). Global Epidemiology of Lung Cancer. Ann Glob Health.

[11] UK National Screening Committee (2022). Targeted screening for lung cancer in individuals at increased risk.

[12] Naidu SB, Wisking A, Karoshi A (2025). Risk Factors Associated With Incidence of Lung Cancer in Never-Smokers: A Systematic Review and Meta-Analysis. *JTO Clin Res Rep*.

[13] Jackson R, Kartoglu I, Stringer C (2018). CogStack - experiences of deploying integrated information retrieval and extraction services in a large National Health Service Foundation Trust hospital. BMC Med Inform Decis Mak.

[14] Kraljevic Z, Searle T, Shek A (2021). Multi-domain clinical natural language processing with MedCAT: The Medical Concept Annotation Toolkit. Artif Intell Med.

