# Supplementary material for: Observational study of predictors and outcomes of lung cancer in never-smokers in the UK (OLIVE): study protocol
Source: BMJ Open Respir Res. 2026 Jun 9;13(1):e003966. doi: 10.1136/bmjresp-2025-003966 (PMC13264864; doi:10.1136/bmjresp-2025-003966)
Supplement: online supplemental file 1 [file bmjresp-13-1-s001.docx]

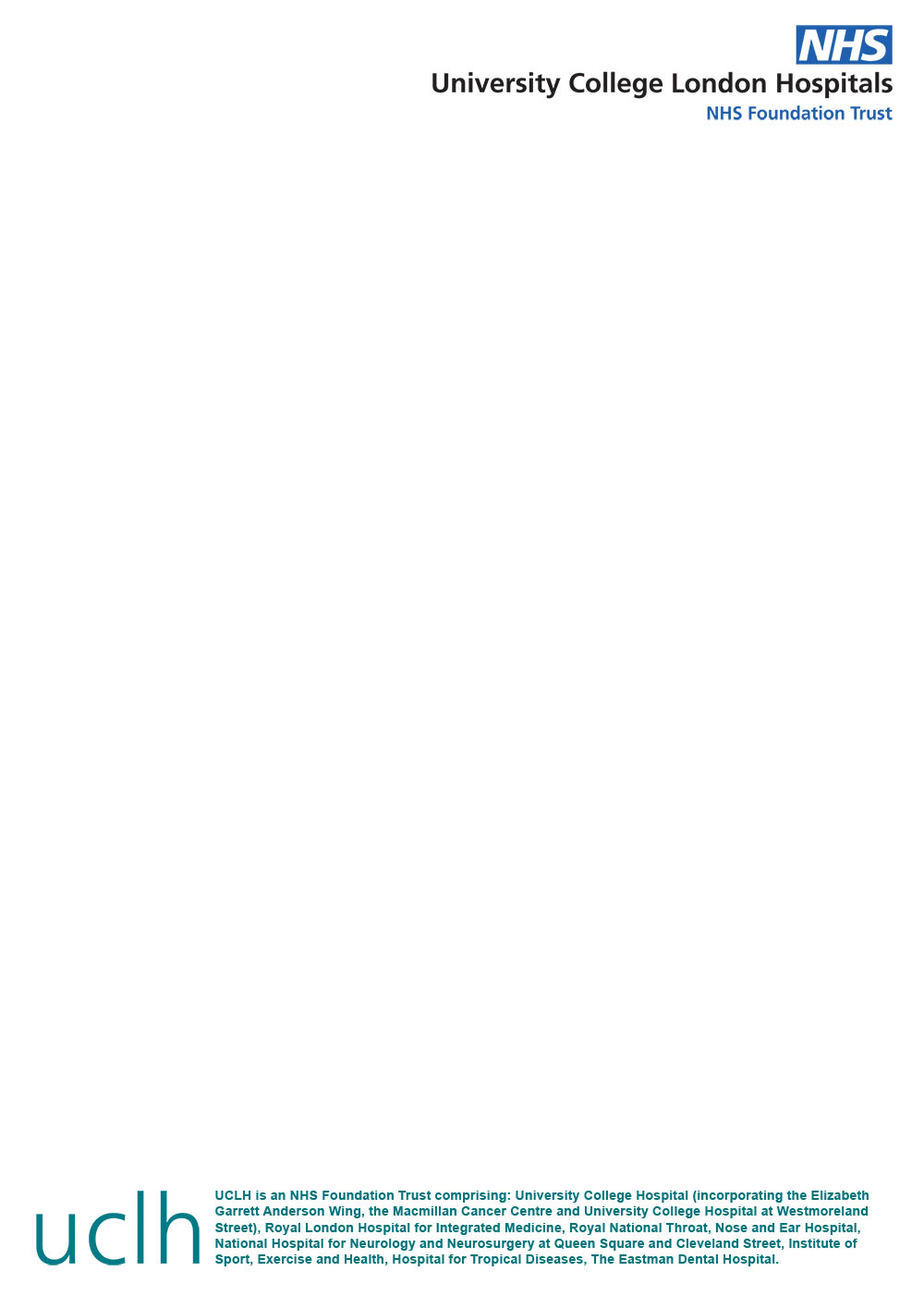

Study Information Sheet
18/07/2024 V1.4

**A study of lung cancer in never smokers in the UK (OLIVE)**

**Summary information**

| **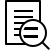** | **What is this study about?** | We are inviting adults, like yourself, who have never smoked and who have been diagnosed with lung cancer to take part in this study.  We are mainly looking at electronic data. This is data that we routinely collect as part of being a patient. |
| --- | --- | --- |
|  |  |  |
| **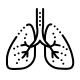** | **Why are we doing this study?** | Lung cancer is common and not all cases are related to smoking. Adults who have never smoked may have different risk factors, symptoms and so on.  We want to improve the diagnosis and management of lung cancer in adults who have never smoked. Finding out these differences will help us. |
|  |  |  |
| **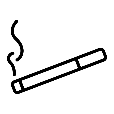** | **What do you mean by ‘never smoked’?** | ‘Never smoked’ means you have smoked less than 100 cigarettes or 75g tobacco (heated) in your lifetime.  It does not include e-cigarettes, cannabis or other non-tobacco substances. |
|  |  |  |
| **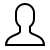** | **What will my involvement be?**  (see p.3 for more) | We will review your electronic health records. There will be no change to your usual clinical care.  In the future, we may ask if we can collect more data (such as measuring pollution in your home or workplace). We will always ask your permission to collect any extra information. |
|  |  |  |
| **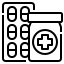** | **Do I have to take part?**  (see p.3 for more) | No. There will be no change to your treatment whether you agree or decline to take part. You can also change your mind about taking part at any time.  If you do not want to take part, just let us or your doctor know. |
|  |  |  |
| **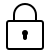** | **What happens to the data that you collect?**  (see p.3-4 for more) | Your data will go through a process to remove certain personal identifiers such as your name. We will store this in a highly secure electronic database. Only researchers with permission can access the data. Your data will always be kept confidential.  We will analyse this data to write reports about lung cancer in those who have never smoked. We will make sure no-one can work out who you are or your identifiable information from these reports. |
|  |  |  |
| 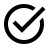 | **What are the benefits and disadvantages of taking part?** | There will be no immediate direct benefit to you. However, we hope to improve the diagnosis and management of lung cancer for future patients.  We do not anticipate any serious risks as we will mainly be looking at your electronic records. We may find information which has an immediate impact on your health. If this happens, we will let you or your clinical team know. We have strict protocols to make sure we act on any other problems that occur. |
|  |  |  |
| **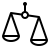** | **Who has approved this study?**  (see p.4 for more) | UCLH is responsible for sponsoring and managing this study across all sites. They have checked that the research meets appropriate standards.  The Confidentiality Advisory Group and Health Research Authority are independent expert groups. They have reviewed our study and legally approved us to access patient records, including those who have passed away.  Our study has also been reviewed by a national ethics committee, a scientific panel and patient representatives. |
|  |  |  |
| 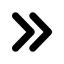 | **What happens next?** | Please read through this information sheet carefully. You can discuss it with your friends or family if you wish. If anything is not clear, or if you have more questions, please ask us.  We will get back in touch with you in a day or two to ask if you have any questions. If you are happy to get involved, we will ask you to sign a consent form electronically or on paper. We will give you a copy of the consent form. We will also put a copy in your medical notes so that your team are aware.  In the future, we will only get in touch if we want to get data that is not already in your electronic notes. |

| 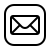 | **How can I find out more?**  (see p.4 for more) | Please contact Dr Sindhu Naidu  Email: [sindhu.naidu@nhs.net](mailto:sindhu.naidu@nhs.net) |
| --- | --- | --- |

**More detailed information**

**
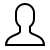
 What will my involvement be?**

We are inviting people who have never smoked, and who have been diagnosed or treated with lung cancer at hospitals which are part of the study. We will be reviewing your GP and hospital electronic health records. There will be no change to your usual clinical care.

The information that we will look at will include:

- information about you (e.g. age, ethnicity)
- your other medical problems
- information about your cancer and treatment

All of this will be kept strictly confidential and analysed only by researchers who have to obey strict rules about keeping your data safe and secure.

In the future, we may ask if we can collect more data (such as measuring pollution in your home or workplace). We will always ask your permission to collect any extra information.

**
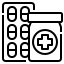
** **Do I have to take part?**

No, taking part in this study is entirely voluntary. There will be no change to your treatment whether you agree or decline to take part.

If you do not want to take part, just let us or your doctor know.

You can change your mind about taking part at any time. We can discuss this whenever you want. You can choose to have ‘no further contact’ with us. This means we will not get in touch to ask you to take part in future research. We will continue to look at and gather data from your electronic health record. You can also choose for us to have ‘no further use’ of your records. This means we cannot collect any more data. However, we will not be able to delete information that is already collected.

We will continue to look at your electronic health records for 5 years. Some people may become sick or confused in this time and may forget they agreed to be included in this study. If this happens, unless you or your legal representative withdraw consent, we will continue to look at your records. We will not invite you for any extra data collection (like blood tests).

**What happens to the data that you collect?**


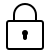


Everyone involved will keep your data safe and secure.

Your data will go through a process to remove some personal identifiers such as your name, contact number and date of birth (‘pseudo-anonymisation’). We will keep some information (like your ethnicity) in our database. We will store your data in a secure electronic database at University College London (UCL). This is protected to a very high standard. Only researchers with permission can access the data.

Your hospital number will be stored in a separate database. Very few researchers will be able to access this. We need to keep this in case we need to contact you or access your electronic health record.

We are required to manage your records in specific ways for the research to be reliable. This means that we won’t be able to let you see or change the data we hold about you.

We will combine many patients’ data to write reports which may be published in scientific journals. We will make sure no-one can work out who you are or your identifiable information from these reports. You will be able to find out about our research and the study results from UCLH’s website and social media. You can also contact us for more information.

Your data will be stored for about 5 years. This is in case we need to check it or for further research. There are strict agreements about who can access this in the future. Your data will not be used to sell you anything. It will not be given to other organisations or companies except for research.

This study is compliant with the requirements of General Data Protection Regulation (GDPR) and the UK Data Protection Act. UCLH is our data controller, which means that they are responsible for looking after your data and making sure it is managed properly.

If you would like more information about how your data is used, please ask your researcher or visit [shorturl.at/tK237](https://d.docs.live.net/3d9c3877fb8ede08/Documents/Research/Never%20smokers/Observational%20UCL/Patient%20information%20materials/shorturl.at/tK237).

**
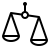
 Who has approved this study?**

UCLH is responsible for sponsoring and managing this study across all sites. They have checked that the research meets appropriate standards. Universities and the NHS are funded from taxes and are expected to do research as part of ‘a task in the public interest’. This is why we are allowed to ask for your data.

The Confidentiality Advisory Group and Health Research Authority are independent expert groups. They have reviewed our study and legally approved us to access patient records, including those who have passed away.

Our study has also been given a favourable opinion by a national research ethics committee. They make sure that this research is in the public interest and meets strict ethical standards. We have also had input from a national scientific panel and a group of patient representatives.

**How can I find out more?**


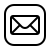


If you, your relatives or your friends have any questions, please contact Dr Sindhu Naidu on [sindhu.naidu@nhs.net](mailto:sindhu.naidu@nhs.net).

If you wish to raise a complaint on how your data is managed, please ask us for the contact details of UCLH’s data protection officer. If you are not satisfied with their response, you can complain to the Information Commissioner’s Office (ICO) (www.ico.org.uk or 0303 123 1113).

If you wish to raise a complaint on any aspect of the study, you can speak to UCLH’s patient advice and liaison service (PALS) on [uclh.pals@nhs.net](mailto:uclh.pals@nhs.net) or 0203 447 3042 or to make a formal complaint [uclh.complaints@nhs.net](mailto:uclh.complaints@nhs.net) or 020 3447 7413.


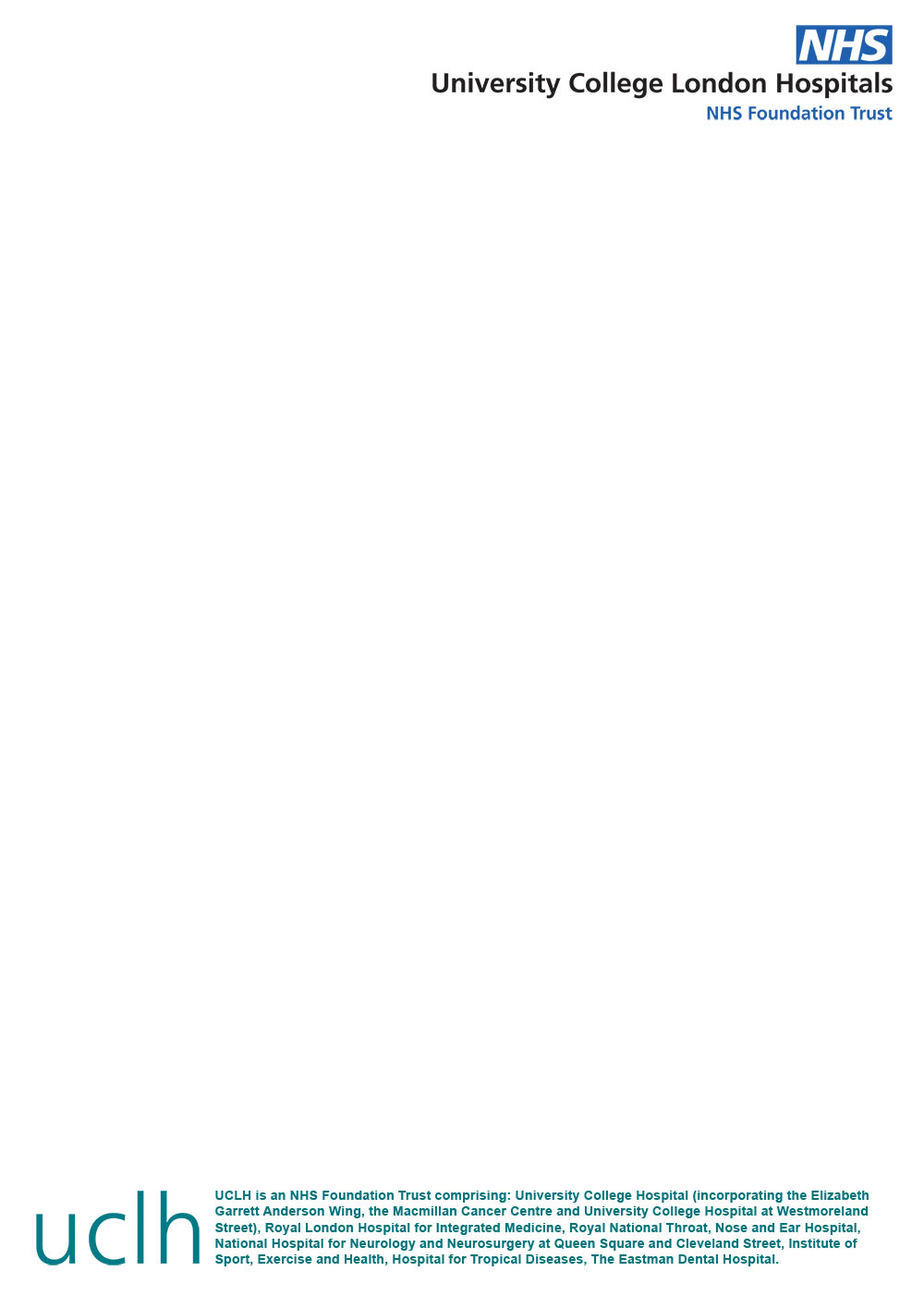


Consent Form
09/08/24 V1.2

**Full title of study:** An observational study of predictors and outcomes of lung cancer in never smokers in the UK

**IRAS ID: 322291**

**Patient MRN:**

|  | **Sign to acknowledge** |
| --- | --- |
| I confirm that I have read the information sheet dated 18/07/24 for the above study. |  |
| I have had the opportunity to consider the information and ask questions. My questions have been answered satisfactorily. |  |
| I understand that my participation is voluntary. I am free to withdraw at any time without giving any reason, without my medical care or legal rights being affected. |  |
| I understand that my electronic health data will be reviewed. Some of my data, with my personal identifiers removed, will be stored in a database for research purposes. |  |
| I consent to my GP data being reviewed for research purposes. |  |
| I understand that if I lose the ability to consent, my electronic data will still be reviewed. I will not be invited for any further data collection. |  |
| I understand that any new information that is found that has an immediate impact on my health will be communicated to me and/or my medical team. |  |
| I understand that the information collected about me will be used to support other research in the future and may be shared anonymously with other researchers. |  |
| I have read this consent form in full. |  |
| I agree to take part in the above study. |  |

__________________ __________________ __________________

Name of participant Signature Date

__________________ __________________ __________________

Name of researcher Signature Da
